# Decision regret in patients with head-and-neck cancer undergoing radiotherapy

**DOI:** 10.1016/j.ctro.2025.101005

**Published:** 2025-06-27

**Authors:** Anna-Maria Tews, Andreas Hinz, Valentin Magnus, Anna Boide, Anja Mehnert-Theuerkauf, Clemens Seidel, Nils Henrik Nicolay, Alexander Rühle

**Affiliations:** a Department of Radiation Oncology, University Medical Center Leipzig, Leipzig, Germany; b Comprehensive Cancer Center Central Germany, Partner Site Leipzig, Leipzig, Germany; c Department of Medical Psychology and Medical Sociology, University Medical Center Leipzig, Leipzig, Germany

**Keywords:** Radiotherapy, Chemotherapy, Surgery, Head-and-neck cancer, Decision regret, Psycho-oncology, Fear of cancer progression, Quality of life

## Abstract

**Purpose:**

Head-and-neck cancer patients often face decisions between treatment options with similar oncological outcomes. In this context, decision regret becomes a central concern, particularly within the framework of shared decision-making. This study aims to assess the prevalence of decision regret and identify associated risk factors among head-and-neck cancer patients.

**Methods:**

This cross-sectional observational study included patients who underwent curative radiotherapy for head-and-neck cancer. Decision regret was assessed using the Decision Regret Scale (DRS). Additional patient-reported outcomes included health-related quality of life (HRQoL), psychological distress, anxiety, depression, and fear of cancer progression (FoP).

**Results:**

Of 300 eligible patients, 192 participated and filled out the DRS. The median age was 59 years (IQR, 45–65), and the median time between the end of radiotherapy and study participation was 45.5 months (IQR, 24–68.5). No decision regret (0 points) in terms of radiotherapy was reported by 27 %, mild regret (1–25 points) by 34 %, and high regret (>25 points) by 39 %. The mean (SD) DRS score regarding radiotherapy, systemic treatment, and surgery was 23.6 (22.4), 20.8 (19.3), and 20.9 (22.2), respectively. Decision regret was associated with HRQoL (*r* = -0.34, *p* < 0.001), distress (*r* = 0.25, *p* < 0.001), anxiety (*r* = 0.20, *p* < 0.01), depression (*r* = 0.19, *p* < 0.01), and FoP (*r* = 0.29, *p* < 0.001). In the multiple regression, a poorer ECOG status (β = 0.18, *p* < 0.01), lower HRQoL (β = -0.22, *p* < 0.01), and higher FoP (β = 0.19, *p* < 0.05) were associated with decision regret.

**Conclusions:**

More than one-third of head-and-neck cancer patients report high decision regret. These findings highlight the need for enhanced shared decision-making to mitigate decision regret in head-and-neck oncology.

## Highlights

• More than one-third of head-and-neck cancer patients reported high decision regret.
• Decision regret regarding radiotherapy, chemotherapy, and surgery was comparable.
• Issues with speech, teeth, and body image were associated with higher regret.
• In the univariate analysis, cancer recurrence was linked to higher decision regret.
• ECOG, HRQoL, and FoP were independently associated with higher decision regret.

## Introduction

Treatment decisions in oncology are complex, particularly for patients with head-and-neck cancer, where multiple modalities including surgery, radiotherapy, chemotherapy, and immunotherapy are available [1,2]. For distinct subsites such as oropharyngeal and laryngeal cancer, oncological outcomes are comparable between different treatment approaches, whereas adverse effects and functional outcomes differ [[3], [4], [5]]. These decisions are often made under considerable uncertainty and time pressure, as treatment must balance oncologic control with functional preservation and health-related quality of life (HRQoL). While shared decision-making has been increasingly emphasized in cancer care, decision regret remains an important yet understudied outcome in this patient population [6].

Decision regret is defined as distress or dissatisfaction with a past healthcare decision, often arising when patients perceive that alternative choices might have led to better outcomes [7,8]. In the oncologic setting, decision regret can be influenced by multiple factors, including treatment-related toxicities, disease progression, psychological distress, and perceived involvement in the decision-making process [[9], [10], [11]]. Recent research suggests that decision regret is inversely correlated with HRQoL and associated with psychological distress and anxiety [12]. However, a critical gap in the literature remains, which is that most studies including head-and-neck cancer patients have evaluated decision regret as a general concept across treatment approaches, without specifically distinguishing between different therapeutic modalities [13]. Given the vastly different side effects and long-term consequences of surgery, radiotherapy, and systemic therapy in head-and-neck cancer patients, it is crucial to assess how decision regret varies by treatment type [14]. Considering the long-term functional side effects associated with radiotherapy, such as dysphagia, xerostomia, and altered speech, patients with head-and-neck cancer may experience substantial regret if these toxicities significantly impact their daily lives [15]. A more granular understanding of decision regret in relation to specific therapies could provide valuable insights for optimizing patient counseling, enhancing shared-decision making, and ultimately reducing decision regret.

To address these gaps, this study aimed to assess the prevalence of decision regret for each treatment component (i.e., radiotherapy, systemic treatment, and surgery) separately in head-and-neck cancer patients treated with either primary radiotherapy, primary chemoradiation, or surgery followed by risk-adapted (chemo)radiation. In addition, we aimed to reveal potential associations between decision regret and HRQoL, distress, anxiety, depression, and fear of cancer progression (FoP).

## Methods

### Study design

This cross-sectional observational study was conducted at the Department of Radiation Oncology, University Medical Center Leipzig, Germany. Inclusion criteria were (i) curative radiotherapy for head-and-neck cancer between 2005 and 2023, (ii) age of ≥ 18 years at the time of radiotherapy, (iii) ability to understand the German questionnaires, and (iv) informed consent to participate in the study. The local ethics committee approved the study in advance (reference numbers: 372/23-ek and 077/23-ek). The study was conducted in compliance with the Declaration of Helsinki, and all patients provided written informed consent. It followed the STROBE guidelines for reporting observational studies.

Between February 2024 and January 2025, potential participants were contacted by telephone and invited to participate in the study. Those who agreed received the informed consent form and paper questionnaires by mail. Patient and treatment characteristics were retrospectively collected from electronic medical records. The reported TNM staging is based on the 7th edition of the UICC TNM classification. Comorbidities were quantified using the Charlson Comorbidity Index, including both patient age and head-and-neck cancer itself [16].

### Questionnaires

Decision regret was assessed using the 5-item Decision Regret Scale (DRS) [17]. Regret was evaluated separately for radiotherapy and, if applicable, concomitant systemic treatment and/or initial surgical resection. The level of decision regret was categorized as absent (0 points), mild (1–25 points), and strong (>25 points), following the previous validation study of the DRS [7]. If patients complete 3–4 items, the mean value of the completed items was used for imputation. HRQoL was measured with the European Organisation for Research and Treatment of Cancer (EORTC) QLQ-C30 questionnaire [18]. We assessed patient-reported outcomes using the EORTC QLQ-HN43 questionnaire, which allows for a nuanced understanding of subjective symptom burden and HRQoL. Distress was assessed with the German version of the National Comprehensive Cancer Network (NCCN) distress thermometer ranging from 0 (no distress) to 10 (extreme distress) [19]. Anxiety and depression were assessed with a single-item thermometer that ranged from 0 (no anxiety/depression) to 10 (extreme anxiety/depression). FoP was quantified with the 12-item version of the Fear of Progression (FoP-Q-12) questionnaire [20], the short-form version of the original 43-item Fear of Progression Questionnaire (FoP-Q) [21]. The 12 items are rated on a five-point Likert scale (1–5), yielding a total score between 12 and 60, with scores of ≥ 34 indicating dysfunctional FoP [22].

### Statistical analysis

Statistical analyses were performed using IBM SPSS Statistics version 29 (IBM Corp., Armonk, NY, USA). Figures were created using GraphPad Prism v10.2.3 (GraphPad Software, Boston, MA, USA). Descriptive statistics summarized the study cohort, including measures of central tendency (mean, median) and dispersion (standard deviation [SD], interquartile range [IQR]) for continuous variables. Univariate associations between decision regret and covariates were examined using Spearman correlations, Pearson correlations and one-way analysis of variance (ANOVA), depending on the scale of each covariate. Internal consistency of the 5-item DRS was calculated using Cronbach’s α. In the linear regression analysis, a listwise deletion of missing data was performed. All clinical parameters that were significantly associated with decision regret in the univariate analysis or were moderately (defined by r ≥ 0.25) correlated with decision regret were simultaneously entered into a linear regression model with decision regret as dependent variable. To avoid collinearity, the functional scales were not entered into the model.

A significance level of *p* < 0.05 was applied to all analyses. As the analyses were exploratory in nature, correction for multiple testing was not performed.

## Results

### Study cohort

**Fig. 1:**
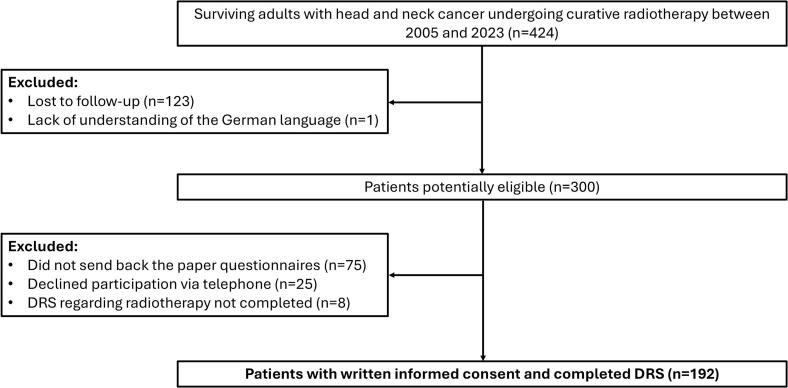
**Study flow diagram.** DRS, decision regret scale.

A total of 300 patients met the inclusion criteria and were deemed potentially eligible for the study. Of these, 200 (67 %) consented to participate and filled out the questionnaires (Fig. 1). Eight participants did not fill out at least 3 items of the DRS regarding their radiotherapy, so that they were excluded from the final analysis, resulting in a final number of 192 patients.

Patient and treatment characteristics of participants and non-responders are summarized in Table 1 and Supplementary **Table S1**. In the study cohort, the median age at the start of radiotherapy was 59 years (IQR, 45–65), and the majority was male (n = 133, 69 %). Most patients (n = 141, 73 %) received surgery followed by adjuvant radiotherapy, while 51 patients (27 %) underwent definitive radiotherapy. Concomitant systemic therapy was administered to 107 patients (56 %). Of those 107 patients, 48 patients (45 %) received systemic treatment as part of definitive chemoradiation, while the remaining 59 (55 %) were treated with postoperative chemoradiation. At the time of this study, the median time interval since the end of the first course of radiotherapy amounted to 45.5 months (IQR, 24–68.5). Cancer recurrence, either local/locoregional or distant, occurred in 37 patients (19 %). The mean HRQoL score (scale “general health/QoL” of the EORTC QLQ-C30) was 60.7 (SD, 21.2).

**Table 1:** **Patient and treatment characteristics (n = 192).** Numbers may not add up to 100 % due to rounding.

		Median (IQR)	
Age at start of first radiotherapy course [years]		59 (45–65)	
Radiotherapy treatment fractions		32 (30–33)	
Total radiation dose [Gy]		64.0 (60.0–69.8)	
Time between last fraction of radiotherapy and study participation [months]		45.5 (24–68.5)	
		**n**	**%**
Gender	Male	133	69
	Female	59	31
Smoking status at first diagnosis	Never smoker	58	30
	Former/current smoker	131	68
	Unknown	3	2
Performance status at the start of radiotherapy	ECOG 0	104	54
	ECOG 1	76	40
	ECOG 2	8	4
	Unknown	4	2
Tumor localization	Oral cavity	51	27
	Nasopharynx	4	2
	Oropharynx	73	38
	Hypopharynx	11	6
	Multilevel pharynx	8	4
	Larynx	23	12
	Salivary gland	10	5
	Nasal cavity/paranasal sinus	8	4
	Cancer of unknown primary in the head-and-neck	4	2
p16 status	Positive	53	28
	Negative	59	31
	Not tested	80	42
T stage	Tx/T0^a^	5	3
	T1	26	14
	T2	73	38
	T3	51	27
	T4	35	18
	Ann Arbor stage IA marginal zone lymphoma	1	1
	Kadish Stage II esthenioneuroblastoma	1	1
N stage	N0	61	32
	N1-3	131	68
Type of radiotherapy	Definitive	51	27
	Adjuvant	141	73
Concomitant systemic treatment	Concomitant systemic treatment	107	56
	No concomictant systemic treatment	85	44
Type of treatment	Definitive radiotherapy	3	2
	Definitive chemoradiation	48	25
	Surgery with postoperative radiotherapy	82	43
	Surgery with postoperative chemoradiation	59	31
Type of systemic treatment (n = 107)	Cisplatin	85	79
	Cisplatin plus 5-fluorouracil	11	10
	Carboplatin plus pembrolizumab^b^	3	3
	Cisplatin plus etoposide	2	2
	Carboplatin plus paclitaxel	2	2
	Cetuximab	2	2
	Mitomycin + 5-fluorouracil	1	1
	Pembrolizumab^c^	1	1
Completion of radiotherapy	Radiotherapy completed	191	99
	Radiotherapy not completed	1	1
Cancer recurrence (local or distant) after radiotherapy	No	155	81
	Yes	37	19

Abbreviations: ECOG, Eastern Cooperative Oncology Group; IQR, interquartile range.

a n = 4 cancer of unknown primary in the head-and-neck, n = 1 rpT0N1 oral cavity carcinoma (initial stage was pT1N0).

b As part of a clinical trial.

c In a patient with a mucosal melanoma of the nasal cavity.

### Prevalence of decision regret

**Fig. 2:**
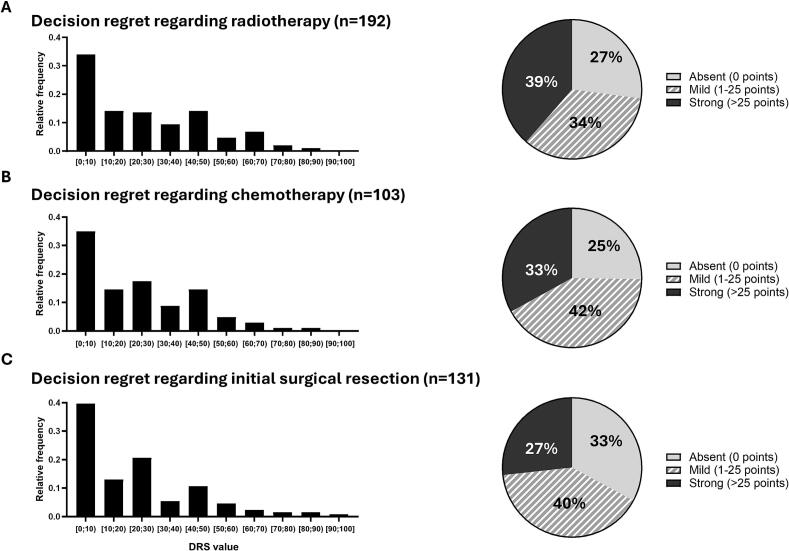
**Frequency of decision regret regarding radiotherapy (A), concomitant systemic treatment (B), and surgery (C).** Histogram showing absolute frequencies regarding the level of decision regret, as assessed using the 5-item Decision Regret Scale (DRS). Pie chart showing the frequency of absent (0 points), mild (1–25 points), and strong (>25 points) decision regret. Square brackets indicate that the value is included, while parentheses mean that the value is excluded. For example, [0,10) represents the range from 0 to 9, including 0 but excluding 10.

The mean value on the DRS regarding radiotherapy treatment was 23.6 (SD, 22.4) points. No decision regret (0 points) in terms of the radiotherapy treatment was reported by 52 patients (27 %), mild regret (1–25 points) by 66 patients (34 %), and high regret (>25 points) by 74 patients (39 %) (Fig. 2). Cronbach’s α of the 5-item DRS was 0.75 in this cohort. The mean value of each single item is provided in the Supplementary **Table S2**.

Of the 107 patients who had been treated with concomitant systemic treatment, 103 (96 %) filled out the DRS regarding concomitant systemic treatment. Here, the mean value on the DRS was 20.8 (SD, 19.3) points. Twenty-six (25 %) reported no regret concerning the choice of concomitant systemic treatment, while 43 indicated mild (42 %) and 34 (33 %) strong regret. One-hundred thirty-one patients (93 %) out of the 141 patients who were treated with initial surgical resection completed the DRS regarding surgery. Mean DRS value was 20.9 (SD, 22.2) points; 43 (33 %) reported no decision regret regarding surgery, whereas 53 (40 %) indicated mild, and 35 (27 %) strong decision regret.

We also performed an analysis limited to patients who received all three treatments (n = 69; 52 with surgery plus adjuvant chemoradiation, 17 with initial surgery followed by definitive chemoradiation for recurrence) in order to ensure a fair comparison. Here, the mean values for decision regret were relatively similar: The mean DRS score regarding radiotherapy, systemic treatment, and surgery were 23.0 (SD, 19.9), 24.3 (SD, 19.6), and 24.5 (SD, 23.7) points, respectively. In this subgroup, patients' DRS showed strong correlations between the different treatment modalities: the strongest correlation was between radiotherapy and systemic treatment (*r* = 0.80, *p* < 0.001), followed by radiotherapy and surgery (*r* = 0.62, *p* < 0.001), and systemic treatment and surgery (*r* = 0.52, *p* < 0.001).

### Association of decision regret with patient-reported outcomes and clinical variables

Among the patient-reported outcome measures analyzed, HRQoL (*r* = -0.34, *p* < 0.001) was inversely correlated with decision regret (Table 2). Speech problems (*r* = 0.40, *p* < 0.001), teeth problems (*r* = 0.39, *p* < 0.001), body image (*r* = 0.37, *p* < 0.001), and social contact (*r* = 0.35, *p* < 0.001), were the four symptom scales that were correlated with decision regret regarding radiotherapy and exhibited a Pearson’s *r* of > 0.3. Distress (*r* = 0.25, *p* < 0.001), anxiety (*r* = 0.20, *p* < 0.01), depression (*r* = 0.19, *p* < 0.01), and FoP (*r* = 0.29, *p* < 0.001) were all found to be weakly correlated with decision regret.

**Table 2:** **Correlation between decision regret regarding radiotherapy and other patient-reported outcome measures.** Correlations with r ≥ 0.3 and *p* < 0.05 are shown in bold.

**Variable**	***r***	***p***	**n**
Global health/QoL (EORTC QLQ-C30)	**−0.34**	**<0.001**	192
**EORTC QLQ-HN43**			
Anxiety	**0.32**	**<0.001**	190
Body image	**0.37**	**<0.001**	190
Dry mouth and sticky saliva	0.16	<0.05	190
Pain in the mouth	0.29	<0.001	189
Sexuality	0.23	<0.01	185
Problems with senses	0.19	<0.05	190
Shoulder problems	0.14	0.06	190
Skin problems	0.09	0.20	189
Social eating	0.29	<0.001	188
Speech	**0.40**	**<0.001**	190
Swallowing	0.26	<0.001	189
Problems with teeth	**0.39**	**<0.001**	189
Coughing	0.11	0.12	190
Swelling in the neck	0.21	<0.01	190
Neurological problems	0.14	0.06	189
Opening mouth	0.20	<0.01	189
Social contact	**0.35**	**<0.001**	190
Weight loss	0.23	<0.01	190
Wound healing	0.26	<0.001	190
Distress	0.25	<0.001	191
Anxiety	0.20	<0.01	191
Depression	0.19	<0.01	191
FoP	0.29	<0.001	192

Abbreviations: FoP, fear of cancer progression; HRQoL, health-related quality of life.

Among the analyzed continuous clinical covariables, age at radiotherapy (*r* = 0.01, *p* = 0.94), Charlson Comorbidity Index (*r* = -0.01, *p* = 0.92) number of radiotherapy fractions (*r* = 0.06, *p* = 0.38), and time between end of radiotherapy and study participation (*r* = -0.02, *p* = 0.78) were not associated with decision regret.

Univariate analyses to explore potential associations of decision regret and covariables were performed per one-way ANOVA. Gender, insurance status, smoking history, tumor localization, p16 status, T stage, N stage, type of radiotherapy or treatment, usage of concomitant systemic treatment, and occurrence of radiotherapy-induced chronic high-grade toxicities were not significantly associated with decision regret regarding radiotherapy treatment (Table 3). The mean DRS regarding radiotherapy was 23.5 (SD, 23.3) for patients undergoing definitive chemoradiation, 22.0 (SD, 21.8) for patients receiving surgery followed by adjuvant radiotherapy, and 24.8 (SD, 21.8) for patients treated with surgery followed by postoperative chemoradiation (*p* = 0.41). However, a reduced ECOG performance status at time of radiotherapy (*p* < 0.05) and cancer recurrence after radiotherapy (*p* < 0.05) were found to be associated with more severe decision regret.

**Table 3:** **Association of decision regret regarding radiotherapy and categorical independent variables per one-way ANOVA (n = 192).** Decision regret regarding radiotherapy treatment was assessed with the 5-item Decision Regret Scale (DRS).

		n	Mean	SD	*p*
Gender	Male	133	22.9	21.1	
	Female	59	25.3	25.2	0.50
Smoking status	Never smoker	58	24.0	22.9	
	Former/current smoker	131	23.9	22.3	0.98
Performance status at the start of radiotherapy	ECOG 0	104	19.6	20.1	
	ECOG 1	76	28.7	24.5	
	ECOG 2	8	26.9	20.2	**<0.05**
Tumor localization	Oral cavity	51	24.7	24.3	
	Nasopharynx	4	23.8	27.5	
	Oropharynx	73	23.9	21.3	
	Hypopharynx	11	21.4	25.8	
	Multilevel pharynx	8	37.5	24.3	
	Larynx	23	24.1	22.9	
	Salivary gland	10	13.5	17.8	
	Nasal cavity/paranasal sinus	8	21.3	24.3	
	CUP in the head-and-neck	4	8.8	14.4	0.52
p16 status	Positive	53	24.3	21.9	
	Negative	59	24.2	23.3	
	Not tested	80	22.6	22.3	0.88
T stage	T0-2	104	22.3	20.8	
	T3-4	86	25.6	24.3	0.31
N stage	N0	61	25.5	24.0	
	N1-3	131	22.7	21.6	0.43
Type of radiotherapy	Definitive	51	24.7	24.3	
	Adjuvant	141	23.2	21.8	0.68
Type of treatment	Definitive radiotherapy	3	43.3	37.5	
	Definitive chemoradiation	48	23.5	23.3	
	Surgery with postoperative radiotherapy	82	22.0	21.8	
	Surgery with postoperative chemoradiation	59	24.8	21.8	0.41
Concomitant systemic treatment	Concomitant systemic treatment	107	24.3	22.4	
	No concomictant systemic treatment	85	22.8	22.6	0.65
Cancer recurrence after radiotherapy	No	155	21.8	21.7	
	Yes	37	31.1	24.0	**<0.05**

Abbreviations: CUP, cancer of unknown primary; ECOG, Eastern Cooperative Oncology Group; SD, standard deviation.

The linear regression model (n = 187), in which ECOG performance status, cancer recurrence, HRQoL, distress, and FoP were simultaneously entered, significantly predicted decision regret (F(5,181) = 8.445, *p* < 0.001) (Table 4). A poorer ECOG performance status (β = 0.18, *p* < 0.01), lower HRQoL (β = -0.22, *p* < 0.01), and higher FoP (β = 0.19, *p* < 0.05) were associated with stronger decision regret.

**Table 4:** **Predictors of decision regret per multiple linear regression analysis.** Complete-case analysis (n = 187) with decision regret as dependent variable.

Variable	β	B	Lower 95 % CI	Upper 95 % CI	*p*
ECOG	0.18	6.90	1.77	12.03	**<0.01**
Cancer recurrence	0.11	6.00	−1.58	13.58	0.12
HRQoL	−0.22	−0.23	−0.39	−0.07	**<0.01**
Distress	0.04	0.30	−0.97	1.57	0.64
FoP	0.19	0.42	0.07	0.77	**<0.05**

Abbreviations: CI, confidence interval; FoP, fear of cancer progression; ECOG, Eastern Cooperative Oncology Group; HRQoL, health-related quality of life.

## Discussion

In this cross-sectional observational study conducted at a large comprehensive cancer center, we observed a notable prevalence of decision regret among head-and-neck cancer patients. Our findings suggest that decision regret is a common experience, with more than one third of patients reporting high levels of regret. Although decision regret was observed across all treatment modalities, the extent of regret was not significantly different between radiotherapy, systemic therapy, and surgery in our study. In the multiple regression analysis, a poorer ECOG performance status, lower HRQoL, and increased FoP were independent predictors for higher decision regret.

In a systematic review and *meta*-analysis, Heirman et al. included a total of 28 studies, with 16 studies contributing to *meta*-analyses on the prevalence of decision regret in head-and-neck cancer patients [13]. The pooled prevalence of clinically significant decision regret, defined as scores above the cutoff on validated questionnaires was 71 %. Another systemic review evaluated only studies that reported decision regret after surgical procedures in the head-and-neck region [23]. In four of the five studies in which head-and-neck cancer patients were included, over a quarter of patients described moderate to strong regret following surgical treatments. The lowest levels of decision regret were reported in a study on transoral robotic surgery, where only 15 % stated moderate to strong regret [24]. In line with our findings, most studies that reported individual DRS item scores found that the statement “The choice did me a lot of harm” consistently elicited the highest levels of decisional regret [[24], [25], [26]].

We observed a significant association between HRQoL and decision regret, which is in line with previous studies with head-and-neck cancer patients [11,12]. While our study cannot establish a causal link, it is plausible that higher decision regret could lead to reduced HRQoL and *vice versa*. Decision regret is often accompanied by emotions such as guilt, disappointment, or frustration, which can negatively impact mental health, potentially leading to stress, anxiety, or depression [12,23]. In turn, a reduced HRQoL could contribute to a more negative, depressive reflection on past treatment decisions, thereby intensifying decision regret. In a prospective cohort study with surgically treated head-and-neck cancer patients, Thomas et al. found that moderate to severe decision regret was associated with baseline frailty, higher depression scores, reduced instrumental activities of daily living, and lower psychological well-being [27]. While distress was found to be associated with psychological well-being and social functioning, little is known about a potential association with decision regret [28,29]. Though distress was moderately correlated with decision regret, it did not remain an independent variable within the multiple regression analysis in our study.

We observed moderate correlations between patient-reported speech problems, teeth problems, and body image with decision regret regarding radiotherapy. In line with this, Shuman et al. found that surviving laryngeal cancer patients who reported worse voice-related QoL experienced more decisional regret [30]. Goepfert et al. found in their study with oropharyngeal cancer survivors that patient-reported dry mouth, difficulty swallowing, teeth problems, and coughing were most strongly correlated with decision regret [25], while Köksal et al. identified five specific post-therapy symptoms (restriction in everyday life, trouble speaking, trouble swallowing, pain in irradiated areas, dissatisfaction with appearance) associated with higher decision regret [10].

ECOG performance status at the time of radiotherapy was independently associated with decision regret, with higher regret reported among patients with poorer performance status. This aligns with our pilot study, in which the German version of the DRS was validated in a general cohort of cancer patients who had undergone radiotherapy [17]. Since data on ECOG performance status at the time of study participation are unavailable, we are unable to determine whether the observed association remains valid at later time points. Longitudinal studies are needed to explore this aspect in greater detail.

To our knowledge, our study is the first that demonstrated a significant association between FoP and decision regret in head-and-neck cancer patients. Hypothetically, higher levels of FoP may contribute to increased decision regret, as patients with high FoP might believe that choosing a different treatment could have lowered their risk of recurrence. Köksal et al. found a moderate correlation between “worry about future health” (which is not exactly the same as FoP) and decision regret (ϱ=0.481, *p* < 0.001) in their study with patients undergoing surgery and adjuvant radiotherapy [10]. The existing evidence regarding the association between FoP and decision regret mainly stems from prostate cancer patients: In a study with 1,229 prostate cancer survivors, more decisional regret and lower satisfaction with information received were associated with higher FoP [31]. Another study also observed that higher FoP was associated with increased decision regret in men with prostate cancer [32].

In contrast to the findings of our study, Windon et al. reported an increase in the DRS of 13 points with each additional treatment modality [9]. In their study, decision regret was significantly higher among patients who underwent surgery with adjuvant radiotherapy, with or without chemotherapy (DRS, median 25), compared to those who received either surgery or radiotherapy alone (median 0; *p* = 0.007). Additionally, participants who received primary radiotherapy with chemotherapy reported a significantly higher median DRS of 15 compared to those treated with surgery or radiotherapy alone (median 0; *p* < 0.001) [9]. In contrast, Kwon et al. did not observe a difference in the DRS between patients treated with surgery alone and those who received surgery followed by postoperative radiotherapy [24]. In the study by Köksal et al., where all patients received surgery followed by adjuvant radiotherapy, 91.7 % of patients reported no regret regarding their decision to undergo surgery [10]. Consistent with our findings on the strong correlation between DRS for radiotherapy and surgery, Köksal et al. reported that patients who regretted undergoing surgery had higher DRS scores for radiotherapy compared to those who did not regret their surgical decision. Goepfert et al. reported that oropharyngeal cancer survivors who underwent surgery alone had a mean DRS of 1.3, while those who received surgery combined with radiotherapy or systemic therapy had a significantly higher mean DRS of 24.4 [25]. In another retrospective observational study with 225 participants, multimodal treatment including radiotherapy was found to be an independent predictor of higher decision regret [11]. It should be noted that only patients who received radiotherapy were included in our study. As a result, comparisons to patients treated with surgery alone are not possible, and definitive conclusions about the impact of the number of treatment modalities on decision regret cannot be drawn from our study. Furthermore, 73 % of patients received primary surgery followed by risk-adapted adjuvant (chemo)radiation, indicating a predominance of surgically treated patients in our study cohort.

Several limitations of our study should be considered. The cross-sectional design restricts the ability to determine causal relationships between decision regret and its associated factors. Future longitudinal studies are needed to understand how decision regret evolves. For instance, Nallani et al. found that patients with decision regret at 3 months post-treatment often reported similar regret at 6 months. [12]. The study population consisted predominantly of long-term survivors, as indicated by a median time of nearly 4 years since completion of radiotherapy, which limits the generalizability of our findings to patients who are closer to the time of treatment. In addition, while we examined a range of clinical and psychological variables, we did not assess other potential contributors to decision regret such as health literacy, satisfaction with treatment, and social support, which have been identified as important factors in previous research [11,12,17]. We acknowledge that detailed clinical variables such as radiotherapy technique, radiation target volume, and surgical extent (e.g., extent of neck dissection or type of reconstruction) were not included in our dataset, which limits our ability to account for their potential impact on toxicity and treatment-related regret. In addition, due to the cross-sectional and voluntary nature of the study design, selection bias cannot be ruled out. Patients with particularly positive or negative experiences may have been more likely to participate, potentially influencing the observed prevalence of decision regret. Regret regarding foregone options, such as the decision not to undergo surgery, was not captured. As a result, the full spectrum of decisional experiences may not be reflected. Lastly, the attribution of decision regret to specific treatment modalities remains a challenge in head-and-neck oncology. Many patients received multimodal treatment, including surgery, radiotherapy, and systemic therapy, which makes it difficult to fully disentangle which modality the regret primarily refers to, even though separate DRS assessments for the different treatment modalities were performed. Despite these limitations, our findings provide new and relevant data that enhance the current understanding of decision regret in head-and-neck cancer patients treated with radiotherapy.

## Conclusions

This study highlights the high prevalence of decision regret among head-and-neck cancer patients, with about one third reporting strong regret. Decision regret was associated with a poorer ECOG performance status, lower HRQoL, and higher FoP, emphasizing its impact on patient well-being. Future longitudinal studies are needed to better understand the evolution of decision regret in adults with head-and-neck cancer.
