# Supplementary material for: Decision regret in patients with head-and-neck cancer undergoing radiotherapy
Source: Clin Transl Radiat Oncol. 2025 Jun 27;54:101005. doi: 10.1016/j.ctro.2025.101005 (PMC12266478; doi:10.1016/j.ctro.2025.101005)
Supplement: Supplementary Data 1 [file mmc1.docx]

**Supplementary Material**

**Supplementary Table 1. Patient and treatment characteristics of non-responders (n=108).** Numbers may not add up to 100% due to rounding.

|  | | Median (IQR) | |
| --- | --- | --- | --- |
| Age at start of first radiotherapy course [years] | | 62 (55-69) | |
| Radiotherapy treatment fractions | | 32 (28-33) | |
| Total radiation dose [Gy] | | 64.0 (61.3-66.0) | |
| Time between last fraction of radiotherapy and telephone call regarding study participation [months] | | 42 (30-63) | |
|  | | **n** | **%** |
| Gender | Male | 78 | 72 |
|  | Female | 30 | 28 |
| Smoking status at first diagnosis | Never smoker | 29 | 27 |
|  | Former/current smoker | 72 | 67 |
|  | Unknown | 7 | 6 |
| Performance status at the start of radiotherapy | ECOG 0 | 40 | 37 |
|  | ECOG 1 | 56 | 52 |
|  | ECOG 2 | 10 | 9 |
|  | ECOG 3 | 2 | 2 |
| Tumor localization | Oral cavity | 15 | 14 |
|  | Nasopharynx | 0 | 0 |
|  | Oropharynx | 54 | 50 |
|  | Hypopharynx | 7 | 6 |
|  | Multilevel pharynx | 3 | 3 |
|  | Larynx | 13 | 12 |
|  | Salivary gland | 7 | 6 |
|  | Nasal cavity/paranasal sinus | 8 | 7 |
|  | Cancer of unknown primary in the head-and-neck | 1 | 1 |
| p16 status | Positive | 31 | 29 |
|  | Negative | 19 | 18 |
|  | Not tested | 58 | 54 |
| T stage | T0 | 1 | 1 |
|  | T1 | 12 | 11 |
|  | T2 | 29 | 27 |
|  | T3 | 34 | 31 |
|  | T4 | 32 | 30 |
| N stage | N0 | 33 | 31 |
|  | N1-3 | 75 | 69 |
| Type of radiotherapy | Definitive | 32 | 30 |
|  | Adjuvant | 76 | 70 |
| Concomitant systemic treatment | Concomitant systemic treatment | 41 | 38 |
|  | No concomitant systemic treatment | 67 | 62 |
| Type of treatment | Definitive radiotherapy | 10 | 9 |
|  | Definitive chemoradiation | 22 | 20 |
|  | Surgery with postoperative radiotherapy | 57 | 53 |
|  | Surgery with postoperative chemoradiation | 19 | 18 |
| Type of systemic treatment (n=41) | Cisplatin | 37 | 90 |
|  | Cisplatin plus 5-fluorouracil | 2 | 5 |
|  | Carboplatin | 1 | 2 |
|  | Cisplatin plus Pembrolizumab^1^ | 1 | 2 |
| Completion of radiotherapy | Radiotherapy completed | 105 | 97 |
|  | Radiotherapy not completed | 3 | 3 |
| Cancer recurrence (local or distant) after radiotherapy | No | 73 | 67 |
|  | Yes | 25 | 23 |
|  | Unknown | 10 | 9 |

^1^As part of a clinical trial

Abbreviations: ECOG, Eastern Cooperative Oncology Group; IQR, interquartile range.

**Supplementary Table 2. Mean values for each item of the 5-item Decision Regret Scale (DRS) regarding radiotherapy.** A 5-point Likert scale ranging from 1 to 5 was used, with higher values indicating more regret.

| **Statement** | **Mean** | **SD** | **n** |
| --- | --- | --- | --- |
| Q1: It was the right decision | 1.47 | 0.91 | 192 |
| Q2: I regret the choice that was made^1^ | 1.96 | 1.37 | 187 |
| Q3: I would go for the same choice if I had to do it over again | 1.77 | 1.25 | 192 |
| Q4: The choice did me a lot of harm^1^ | 2.90 | 1.63 | 189 |
| Q5: The decision was a wise one | 1.65 | 1.00 | 191 |

^1^ Items 2 and 4 were reverse coded so that higher values indicate more regret. The reverse coded values are shown here.

Abbreviation: SD, standard deviation.
